# Physically Guided Attention Mechanism for Underwater Motion Deblurring via Cepstrum-Based Blur Estimation

**DOI:** 10.3390/jimaging12050186

**Published:** 2026-04-26

**Authors:** Ning Hu, Shuai Li, Jindong Tan

**Affiliations:** 1 Department of Mechanical, Aerospace and Biomedical Engineering, University of Tennessee, Knoxville, TN 37916, USA; tan@utk.edu; 2 Department of Environmental Engineering Sciences, University of Florida, Gainesville, FL 32611, USA; shuai.li@ufl.edu

**Keywords:** underwater image restoration, motion deblurring, blur kernel estimation, cepstrum analysis, vision transformer, point spread function

## Abstract

Underwater images often suffer from mixed degradations, including motion blur, which reduce structural clarity and adversely affect downstream vision tasks. To address this problem, we propose a physically guided Transformer framework for underwater motion deblurring. The proposed method combines two-stage cepstrum-based blur estimation with a point spread function (PSF)-guided self-attention mechanism. Specifically, blur parameters are first robustly estimated through cepstrum analysis, ellipse fitting, and negative-peak refinement, and the resulting PSF is then embedded into the Transformer attention module to guide feature aggregation. On the real underwater benchmark datasets UIEB Challenge-60 and EUVP330, the proposed method achieves UIQM/UCIQE scores of 4.09/0.56 and 3.40/0.58, respectively, significantly outperforming UFPNet and Phaseformer, thereby demonstrating superior perceptual restoration in terms of sharpness, contrast, and color consistency. On the synthetic test set, the proposed method attains 24.23 dB PSNR and 0.918 SSIM, outperforming both recent deep models and classical non-blind deconvolution methods, which confirms its strong restoration fidelity and structural consistency. In the controlled water-tank experiments, the proposed method consistently achieves the best performance under different camera motion speeds, demonstrating excellent robustness and practical applicability. Overall, the proposed framework provides an effective and physically interpretable solution for underwater motion deblurring.

## 1. Introduction

Underwater imaging plays an important role in marine observation, environmental monitoring, infrastructure inspection, and autonomous robotic exploration. However, underwater images are often severely degraded by scattering, absorption, non-uniform illumination, and motion-induced blur. Among these factors, motion blur is particularly detrimental because it directly reduces structural clarity and weakens the visibility of edges and textures. In underwater environments, motion blur is commonly caused by camera motion, relative motion between the camera and observed objects, and the longer exposure times required under low-light conditions [1,2]. As a result, degraded images can negatively affect downstream vision tasks such as object recognition, feature matching, visual odometry, autonomous navigation, and robotic mapping, which are critical for underwater perception systems [3,4].

Image deblurring has been widely studied in computer vision. Traditional approaches are mainly based on analytical formulations, including blind deconvolution, Wiener filtering, and variational Bayesian inference [5,6,7]. These methods usually estimate the blur kernel first and then recover the latent sharp image by deconvolution. Although such model-based methods are physically interpretable, their performance strongly depends on the validity of the assumed blur formation model and on the accuracy of kernel estimation. In realistic underwater scenes, however, dynamic motion, low contrast, weak texture, attenuation, and backscatter often violate these assumptions and make blur estimation much less reliable [8,9]. As a result, conventional analytical methods may produce unstable restoration results when applied to underwater imagery.

With the rapid development of deep learning, convolutional neural network (CNN)-based methods have significantly improved the performance of image deblurring. Representative models such as DeblurGAN, Deep Multi-scale CNN, and MIMO-UNet learn a direct mapping from blurred images to restored images by exploiting large-scale training data [10,11,12]. Compared with traditional deconvolution-based pipelines, these methods can better capture complex image priors and generally achieve stronger restoration quality. Nevertheless, CNN-based architectures mainly rely on local convolution operations, which may limit their ability to model long-range dependencies in heavily blurred regions. This limitation can affect the recovery of extended blur structures, repeated textures, and globally consistent details in underwater images.

More recently, Transformer architectures have shown strong potential for image restoration because self-attention can model long-range interactions more effectively than local convolutions. Methods such as Restormer, Uformer, and MPRFormer have reported promising performance on general image restoration and motion deblurring tasks [13,14,15]. In parallel, several recent studies have explored combining deep restoration networks with physically meaningful priors, indicating that the integration of physical knowledge and data-driven learning can improve robustness in complex imaging scenarios [16,17]. Despite these advances, most existing deblurring networks still treat blur mainly as an implicit degradation to be learned from data. In other words, the physical structure of the blur is rarely estimated explicitly and rarely injected into the feature aggregation process of the network itself.

This limitation becomes more critical in underwater imaging. Unlike many terrestrial deblurring settings, underwater images often suffer from mixed degradations, where motion blur appears together with scattering, absorption, color distortion, and weak local contrast. Moreover, many existing underwater restoration studies focus primarily on color correction, haze removal, or contrast enhancement, while explicit modeling of motion blur remains limited. Consequently, purely data-driven restoration networks may have difficulty adapting to sample-dependent blur patterns in underwater scenes, especially when the blur is directionally structured but the image texture is weak. For such cases, introducing an explicit and physically interpretable blur prior can provide useful guidance beyond what is available from end-to-end learning alone.

To address this problem, this paper proposes a physically guided Transformer-based framework for underwater motion deblurring. The proposed method combines two-stage cepstrum-based blur estimation with PSF-guided self-attention. Specifically, motion blur parameters are first estimated from the degraded underwater image through preprocessing, cepstrum analysis, morphological enhancement, coarse ellipse fitting, and negative-peak refinement. These estimated parameters are then used to construct the corresponding point spread function (PSF), which serves as a compact representation of the blur orientation and blur length. Instead of using this PSF merely as an auxiliary input or a post-processing cue, we embed it into the self-attention module of a Transformer-based restoration network so that feature aggregation can be guided by image-dependent blur structure during restoration.

In summary, the main gain of this work is that it bridges physically interpretable blur estimation and Transformer-based restoration. Rather than relying solely on data-driven attention, the proposed network is guided by sample-specific PSF cues estimated from the observed underwater image. This design allows the model to adapt its feature aggregation to the underlying blur pattern and improves restoration in motion-blur-dominant underwater scenes.

The main contributions of this work are summarized as follows:

(1) A physically guided attention mechanism for underwater motion deblurring is proposed. The estimated PSF is embedded as an image-dependent guidance term in the Transformer self-attention module, enabling blur-aware feature aggregation during restoration rather than using physical priors only as pre-processing or side information.

(2) A two-stage blur parameter estimation strategy is developed for weak-texture underwater images. The method combines cepstrum-domain analysis, morphological processing, ellipse fitting, and negative-peak refinement to improve the estimation of blur orientation and blur length under noisy underwater conditions.

(3) A physically guided Transformer-based restoration framework is constructed and validated on public underwater datasets and controlled water-tank images. The experimental results demonstrate that the proposed method improves structural recovery and color consistency in blurred underwater images and achieves better quantitative performance than recent comparison methods.

The remainder of this paper is organized as follows: Section 2 reviews related work on motion blur modeling, blur estimation, and underwater image restoration. Section 3 presents the proposed method, including the underwater motion blur formulation, the two-stage blur estimation procedure, and the PSF-guided attention mechanism. Section 4 describes the experimental settings and reports quantitative and qualitative results. Section 5 discusses the findings, limitations, and practical implications of the proposed framework. Finally, Section 6 concludes this paper.

## 2. Related Work

### 2.1. Classical Motion Blur Modeling and Blur Estimation

Motion blur restoration is commonly formulated as an inverse problem in which the observed blurred image is modeled as the convolution of a latent sharp image with a blur kernel, usually with an additional noise term [18]. Under this formulation, successful restoration depends heavily on accurate blur kernel estimation followed by effective deconvolution [19,20]. For this reason, classical motion deblurring methods have traditionally focused on estimating the blur kernel as explicitly as possible.

These classical approaches can be broadly divided into non-blind and blind deblurring methods. Non-blind methods assume that the blur kernel is known in advance and then recover the latent image by solving a deconvolution problem. Representative examples include Wiener filtering [6] and the Richardson–Lucy iterative deconvolution algorithm [21]. Such methods are computationally efficient and physically interpretable, but their practical utility is limited because the blur kernel is rarely available in real imaging scenarios [22].

Blind deblurring methods aim to estimate the blur kernel directly from the observed image and are therefore more flexible in practical use. Early studies explored transform-domain and geometric cues to estimate blur direction and scale, including Radon-transform-based analysis [23,24] and cepstrum-based analysis [25]. These methods exploit regular stripe or periodic structures in the frequency or cepstrum domain and can provide interpretable estimates of blur orientation and blur length. However, their performance depends strongly on the visibility of these structures. In scenes with weak textures, low contrast, noise, or very small blur scales, the corresponding frequency-domain signatures can become unstable, which reduces estimation accuracy [26,27].

This limitation is particularly relevant for underwater imaging. Compared with clear in-air scenes, underwater images often exhibit reduced contrast, color attenuation, backscatter, and local noise-like disturbances. These factors make the spectral signatures of motion blur more difficult to detect reliably. Therefore, although classical kernel estimation methods provide an important physical foundation, they require careful adaptation before they can be used robustly in underwater motion deblurring.

### 2.2. Deep Learning for Image Deblurring

With the development of deep learning, image deblurring has increasingly shifted from explicit kernel estimation and deconvolution to end-to-end restoration. Convolutional neural network (CNN)-based methods learn a direct mapping from blurred images to restored images using large-scale training data. Representative examples include DeblurGAN [10], Deep Multi-scale CNN [11], and MIMO-UNet [12]. By learning complex image priors from data, these models usually outperform traditional analytical pipelines in terms of restoration quality and robustness to moderate deviations from ideal blur models.

However, CNN-based deblurring methods mainly rely on local convolution operations. Although multi-scale designs and residual learning improve their receptive field, long-range dependency modeling remains indirect. This can limit their ability to recover globally consistent motion structures, especially in images with extended blur trails, repeated patterns, or large degraded regions. For underwater images, where useful texture is already weakened by optical degradation, this limitation can further reduce restoration performance.

Transformer-based methods address part of this issue by introducing self-attention for long-range feature aggregation. Methods such as Restormer [13], Uformer [14], and MPRFormer [15] have shown strong performance in image restoration and motion deblurring tasks because self-attention can better capture global context than purely local convolution. These methods demonstrate that long-range interaction is important for restoring complex degraded structures. Nevertheless, most Transformer-based deblurring models remain primarily data-driven: the network is trained to infer blur implicitly from data, but the blur formation process itself is not explicitly represented in the model.

More recently, generative and diffusion-based restoration frameworks have also been explored. For example, Image-Conditional Diffusion Transformer (ICDT) combines diffusion modeling with Transformer-based feature extraction [17], while Physics-Aware Diffusion Transformer (PA-Diff) introduces physical priors into diffusion-based restoration. These studies suggest that combining deep generative restoration with physically meaningful guidance can improve robustness in difficult imaging conditions. Even so, in most existing methods, physical priors are used at a relatively general level and are not directly tied to sample-specific motion blur estimation.

From this perspective, a key gap remains between physically interpretable blur estimation and feature aggregation inside deep restoration networks. Existing deep deblurring models are powerful at representation learning, but they often do not explicitly use image-specific blur parameters to guide how features should interact during restoration.

### 2.3. Underwater Image Restoration and Physically Guided Learning

Underwater image restoration has traditionally focused on optical degradations such as scattering, absorption, low contrast, and color distortion. Many studies aim to improve visibility, color balance, and scene contrast under underwater imaging conditions, but explicit modeling of motion blur remains relatively limited. In practice, underwater images often suffer from mixed degradation, where motion blur coexists with attenuation, haze-like backscatter, weak illumination, and color shifts. This makes underwater motion deblurring more challenging than generic image deblurring under standard terrestrial imaging assumptions.

A further difficulty is that blur patterns in underwater scenes are often sample-dependent, while image textures are weak and degraded. In such cases, purely data-driven restoration models may not adapt well to the actual blur structure of a given image. This issue is especially important when the restoration model must recover structural details for downstream tasks such as feature matching, underwater visual SLAM, and robotic navigation.

Recent studies that integrate physical information with deep learning indicate a promising direction for addressing this problem. However, only a limited number of approaches attempt to combine physically estimated blur cues with deep restoration specifically for underwater motion blur. Most existing methods either focus on generic motion deblurring without underwater-specific considerations, or they focus on underwater enhancement without explicitly incorporating motion blur formation cues into the restoration mechanism itself.

The present work is positioned in this gap. Unlike classical methods, the proposed framework does not rely on deconvolution alone after kernel estimation. Unlike purely data-driven CNN or Transformer models, it does not treat blur only as an implicit latent factor to be learned from data. Instead, it first estimates motion blur parameters from the underwater image using a cepstrum-based two-stage estimation strategy designed for weak-texture scenes, and then constructs a point spread function (PSF) from these parameters. The estimated PSF is subsequently injected into the Transformer as an image-dependent guidance term in self-attention. In this way, the proposed method combines the interpretability of physical blur estimation with the representation power of Transformer-based restoration, while explicitly addressing the motion-blur-dominant underwater setting.

Table 1 summarizes representative related work in terms of task type, physical prior usage, explicit blur estimation, underwater specificity, and main limitations.

## 3. Method

**Figure 1:**
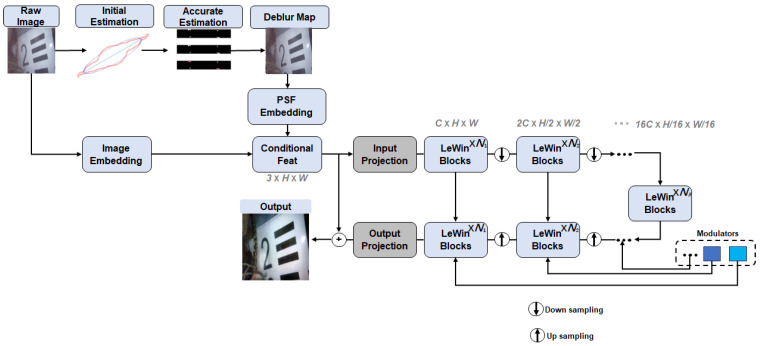
Proposed algorithm overview.

As illustrated in Figure 1, the proposed framework consists of two coupled stages. In the first stage, motion blur parameters are estimated from the degraded underwater image using a physics-based two-stage procedure. The estimated blur length and blur angle are then used to construct the corresponding point spread function (PSF). In the second stage, the estimated PSF is injected into a Transformer-based restoration network to guide feature aggregation during image reconstruction. In contrast with purely data-driven restoration, the proposed framework explicitly estimates sample-specific blur structure and uses it to modulate attention computation.

### 3.1. Underwater Motion Blur Imaging Model and Two-Stage Blur Parameter Estimation

#### 3.1.1. Underwater Motion Blur Imaging Model

Underwater image degradation is jointly affected by optical attenuation, backscatter, and motion blur. Let J(x,λ) denote the latent sharp scene radiance at spatial location x and wavelength λ, let k denote the motion blur kernel (PSF), let t(x,λ)=exp(−β(λ)d(x)) denote the transmission map, let $B_{\infty }(\lambda )$ denote the background light, and let n(x,λ) denote image noise. The observed blurred underwater image $I_{b}(x,\lambda )$ can be expressed as follows:

(1) $$I_{b}\left(x,\lambda \right)=\left(\left(J\left(∙,\lambda \right)*k\right)\left(x\right)\right)t\left(x,\lambda \right)+B_{\infty }\left(\lambda \right)\left(1-t\left(x,\lambda \right)\right)+n\left(x,\lambda \right)$$

where ∗ denotes convolution.

In this work, we focus on the motion-blur-dominant restoration setting. For locally consistent depth or controlled underwater scenes, the attenuation and backscatter terms can be absorbed into an intermediate degraded underwater image $I_{u}$. The observation model is then approximated as follows:

(2) $$I_{b}\left(x\right)\approx \left(I_{u}*k\right)\left(x\right)+n\left(x\right)$$

where $I_{u}$ denotes the latent underwater image before motion blur. Under this formulation, accurate estimation of the blur kernel k becomes the key step for physically guided restoration. This motivates the first stage of the proposed method, in which blur parameters are estimated explicitly from the input image.

#### 3.1.2. Preprocessing and Cepstrum Extraction

Motion-blurred images generated by camera motion usually exhibit approximately uniform blur within a local crop. Therefore, the input image is first cropped to a square region according to the shorter image side so that blur estimation is performed on a geometrically consistent patch. Let $I_{c}$ denote the cropped image.

To improve the visibility of blur-related stripe structures, three preprocessing operations are applied. First, histogram equalization is used to increase the contrast between bright and dark stripe patterns. Second, a two-dimensional Hanning window is multiplied with the image to suppress boundary truncation artifacts before Fourier transformation. Third, median filtering is used to reduce isolated noise while preserving edge-like structures that are important for frequency-domain analysis. The preprocessed image $I_{p}$ is written as follows:

(3) $$I_{p}\approx M\left(H_{w}⊙H\left(I_{c}\right)\right)$$

where H(⋅) denotes histogram equalization, $H_{w}$ denotes the Hanning window, ⊙ denotes element-wise multiplication, and M(⋅) denotes median filtering.

The Fourier spectrum of $I_{p}$ is then computed, and its dynamic range is compressed to highlight blur-related periodic patterns. The cepstrum representation is obtained as follows:

(4) $$C=F^{-1}\left(\log\left(\left|F\left(I_{p}\right)\right|+\varepsilon \right)\right)$$

where F(⋅) and $F^{-1}(\cdot )$ denote the Fourier transform and inverse Fourier transform, respectively, ∣⋅∣ denotes the spectrum magnitude, and *ε* is a small constant used to avoid numerical instability. The cepstrum domain emphasizes periodic structures related to motion blur and is therefore suitable for blur parameter estimation.

#### 3.1.3. Morphological Enhancement in Cepstrum Structures

The raw cepstrum map may still contain noise, background artifacts, and intersecting structures that interfere with blur estimation. To enhance the dominant blur-related patterns, grayscale enhancement, binarization, and morphological opening are performed sequentially. Let G(⋅), B(⋅), and O(⋅) denote grayscale enhancement, binarization, and morphological opening, respectively. The processed cepstrum map is written as follows:

(5) $$C_{m}=O\left(B\left(G\left(C\right)\right);S_{r}\right)$$

where $S_{r}$ denotes a rectangular structuring element. A rectangular element is used because blur-related cepstrum structures typically appear as elongated streaks. The contours extracted from $C_{m}$ are then used as candidates for subsequent blur estimation.

#### 3.1.4. Two-Stage Estimation of Blur Angle and Blur Length

The blur parameters are estimated by a coarse-to-fine strategy.

In the first stage, a least-squares ellipse is fitted to the dominant cepstrum contour. Let the fitted ellipse be denoted by $E_{0}$, and let α denote the angle between its major axis and the horizontal axis. The major-axis direction provides a coarse estimate of blur orientation, while the ellipse extent provides a coarse estimate of blur length. The coarse estimates are denoted by $\left(L_{0},\theta_{0}\right)$.

In the second stage, the blur parameters are refined using negative peak detection in the cepstrum domain. According to cepstrum characteristics of linear motion blur, two dominant negative peaks are expected to appear approximately symmetrically with respect to the cepstrum center, and the distance between them corresponds to twice the blur length. To improve robustness, the strongest candidate negative peaks are first selected, and only peak pairs satisfying a symmetry constraint and lying within the search region implied by the coarse ellipse are retained.

Let the selected peak pair be $p_{1}=(x_{1},y_{1})$ and $p_{2}=(x_{2},y_{2})$. The refined blur angle is then defined by the direction of the line segment connecting the two peaks:

(6) $$\theta =\mathrm{atan}2\left(y_{2}-y_{1},x_{2}-x_{1}\right)$$

and the refined blur length is computed as follows:

(7) $$L=\frac{1}{2}{\left\|p_{2}-p_{1}\right\|}_{2}$$

Here, $∥\cdot {∥}_{2}$ denotes the Euclidean norm. Therefore, L represents the final blur length and θ represents the final blur angle. Compared with single-stage estimation, this two-stage design improves robustness in weak-texture underwater scenes by combining global structural cues from ellipse fitting with local peak cues from cepstrum refinement.

#### 3.1.5. PSF Construction

After estimating L and θ, a linear motion PSF P is constructed. The PSF is centered in a discrete kernel support and aligned with angle θ. Its nonzero values lie on a line segment of length L, and the kernel is normalized so that:

(8) $$\sum_{u,v}P\left(u,v\right)=1$$

The constructed PSF serves as a compact physical representation of sample-specific blur structure and is subsequently used to guide the restoration network.

### 3.2. PSF-Guided Attention for Transformer-Based Restoration

After blur estimation, the restoration stage takes the degraded image together with the estimated PSF as input. A modified Uformer architecture is adopted as the restoration backbone because its window-based self-attention can capture long-range dependencies that are important for image deblurring. Unlike standard Transformer restoration, the proposed method explicitly injects the estimated PSF into the attention computation so that feature aggregation is adapted to the blur structure of the current sample.

#### 3.2.1. PSF Representation for Attention Guidance

Let the estimated PSF be denoted by $P\in R^{k\times k}$, where k is the PSF kernel size. To match the local attention resolution, P is resized to the Transformer window size $w_{s}\times w_{s}$. The resized PSF is denoted by:

(9) $$P_{r}=\mathrm{Resize}\left(P,w_{s},w_{s}\right)$$

Let $N=w_{s}^{2}$ denote the number of tokens in one attention window. The resized PSF is flattened into a guidance vector:

(10) $$g=\mathrm{vec}\left(P_{r}\right)\in R^{N}$$

where vec(⋅) denotes vectorization. The vector g encodes the blur orientation and blur extent of the current image in a compact form.

#### 3.2.2. PSF-Guided Window-Based Self-Attention

For one local window, let the input feature matrix be $X_{w}\in R^{N\times d}$, where d is the channel dimension. For the *h*-th attention head, the query, key, and value matrices are computed in the standard way:

(11) $$Q_{h}=X_{w}W_{h}^{Q},K_{h}=X_{w}W_{h}^{K},V_{h}=X_{w}W_{h}^{V}$$

where $W_{h}^{Q}$, $W_{h}^{K}$, and $W_{h}^{V}$ are learnable projection matrices.

To inject blur information into attention, the PSF guidance vector g is transformed into a head-specific spatial bias matrix:

(12) $$B_{h}=R_{h}\left(g\right)\in R^{N\times N}$$

where $R_{h}(\cdot )$ denotes a learnable projection-and-reshape operation. Therefore, $B_{h}$ is an image-dependent spatial bias derived from the estimated PSF.

The proposed PSF-guided attention is defined as follows:

(13) $${\mathrm{Attn}}_{h}\left(X_{w},g\right)=\mathrm{Softmax}\left(\frac{Q_{h}K_{h}^{T}}{\sqrt{d_{h}}}+\gamma_{h}B_{h}\right)V_{h}$$

where $d_{h}$ denotes the feature dimension of one attention head and $\gamma_{h}$ is a learnable scalar controlling the strength of PSF guidance.

Compared with direct concatenation of PSF features and image features, Equation (13) allows the estimated blur prior to explicitly modify token-to-token affinity during feature aggregation. In this way, the network does not merely receive blur information as an auxiliary input; instead, it uses the PSF to influence how information is propagated within each attention window. This is especially beneficial for motion deblurring because the degradation is directional and structurally related to long-range dependency modeling.

#### 3.2.3. Interpretation of the Guidance Term

The proposed guidance term differs from conventional relative positional bias. Relative positional bias encodes fixed geometric offsets between tokens and is shared across all inputs. In contrast, the proposed $B_{h}$ is estimated from the PSF of the current image and therefore depends on the sample-specific degradation. This makes the attention bias physically meaningful and image-adaptive.

After multi-head PSF-guided attention is computed, the outputs from all heads are concatenated and passed through the remaining Uformer blocks in the standard hierarchical encoder-decoder manner. Through this design, the restoration network combines the representation power of Transformer-based restoration with explicit physical guidance derived from blur estimation.

### 3.3. Training Objective

The restoration network is trained in a supervised manner using a combination of pixel reconstruction loss and structural similarity loss:

(14) $$L_{\mathrm{rest}}={\left\|I_{\mathrm{pred}}-I_{\mathrm{ref}}\right\|}_{1}+\lambda_{\mathrm{ssim}}\left(1-\mathrm{SSIM}\left(I_{\mathrm{pred}},I_{\mathrm{ref}}\right)\right)$$

where $I_{\mathrm{pred}}$ denotes the restored image, $I_{\mathrm{ref}}$ denotes the reference image, and $\lambda_{\mathrm{ssim}}$ balances pixel accuracy and structural consistency. The $L_{1}$ term is used to preserve sharp edges and reduce oversmoothing, while the SSIM term encourages recovery of structural information.

## 4. Experimental Testing

### 4.1. Datasets and Data Preparation

To comprehensively evaluate the proposed physically guided Transformer framework for underwater motion deblurring, experiments were conducted on public underwater benchmarks as well as on a controlled real-world water-tank platform. The public benchmark evaluation used two widely adopted underwater image sets: the UIEB Challenge-60 subset [30], which contains 60 challenging underwater images, and the EUVP330 subset [31], which contains 330 underwater validation images. These two datasets were selected because they cover diverse underwater scenes with different visibility conditions, color distortions, and blur severity, thereby providing representative real-world test cases for underwater restoration. Since the evaluated images in these benchmark subsets do not provide paired sharp ground-truth references, they were used primarily for no-reference quantitative evaluation and qualitative comparison.

Because the restoration network in Section 3.3 is trained in a supervised manner, a paired training set was constructed separately from the benchmark test sets. Specifically, reference underwater images were collected from UIEB, and motion blur was synthetically generated to form blurred–reference training pairs. For each reference image, a linear motion kernel parameterized by blur length L and blur angle θ was sampled to generate a degraded input image. The blur length was randomly sampled from [5,25] pixels, and the blur angle was randomly sampled from [0°, 180°]. In addition, no additional noise was introduced, unless otherwise stated, to better simulate practical underwater imaging conditions. The resulting paired samples were used for supervised training, while the UIEB Challenge-60 and EUVP330 subsets were reserved exclusively for testing in order to avoid overlap between training and evaluation.

For model development, the paired data were divided into training and validation subsets using a ratio of [9:1]. During preprocessing, all images were resized to [256 × 256] before being fed into the network. If a synthetic paired test set was additionally constructed for full-reference evaluation, it was kept fully disjoint from the training split and was used only for reporting full-reference metrics such as PSNR and SSIM. This design separates supervised learning from real-image benchmark evaluation and allows both controlled and practical performance to be examined within a unified framework.

In addition to public benchmark testing, real-world validation was performed using a controlled water-tank platform. The tank scene was arranged with stones, aquatic plants, and sand to simulate weak-texture underwater environments similar to seabed conditions. A monocular camera was mounted on a motion platform and used to capture image sequences during controlled linear and rotational motion, thereby generating realistic underwater motion blur. Water turbidity and illumination were further adjusted by introducing suspended particles and modifying the lighting configuration, which enabled the acquisition of underwater images under different blur levels, visibility conditions, and lighting environments. These real images were used to assess the practical applicability and robustness of the proposed framework beyond public benchmark datasets.

### 4.2. Implementation Details

All experiments were implemented in the PyTorch (version 1.13.1) framework and executed on a workstation equipped with an NVIDIA RTX GPU. The proposed PSF-guided Transformer restoration network was trained for 300 epochs using the Adam optimizer. The initial learning rate was set to 1 × 10^−4^ and was decayed after epoch 250 for fine-tuning. The Adam hyperparameters were set to $\left(\beta_{1},\beta_{2})=(0.9,\;0.999\right)$, with a weight decay of 1 × 10^−4^. The batch size was set to 8. During training, all input images were resized to 256 × 256 pixels. The loss function followed Section 3.3, where the reconstruction objective consisted of an $L_{1}$ term and an SSIM term, and the weighting coefficient $\lambda_{\mathrm{ssim}}$ was empirically set to 0.5. The paired training data were divided into training and validation subsets at a ratio of 9:1.

For each training sample, the restoration network took the degraded underwater image together with the estimated blur kernel as input. The blur kernel, i.e., the point spread function (PSF), was first generated from the blur length and blur angle estimated by the procedure in Section 3.1, and then resized to match the local attention resolution of the Transformer. Specifically, the PSF guidance vector was generated using a window size of 32 × 32, consistent with the attention window size adopted in the Transformer blocks. This design ensured that the physically estimated blur prior could be injected into window-based self-attention in a dimensionally compatible manner. During inference, the same preprocessing and blur estimation pipeline described in Section 3.1 was first applied to the input underwater image to obtain the corresponding PSF. The blurred image and the estimated PSF were then jointly fed into the restoration network to generate the final deblurred result. Unless otherwise stated, all compared learning-based methods were evaluated on the same test images under the same input resolution and metric settings to ensure fair comparison.

### 4.3. Evaluation Metrics

To comprehensively evaluate the proposed method, quantitative assessment was conducted from three aspects: no-reference image quality evaluation on real underwater benchmark images, full-reference restoration evaluation on paired synthetic data, and blur parameter estimation accuracy when ground-truth blur parameters were available.

For real underwater benchmark images without paired sharp references, two widely used no-reference underwater image quality metrics, namely the Underwater Image Quality Measure (UIQM) and the Underwater Color Image Quality Evaluation (UCIQE), were adopted. UIQM evaluates underwater image quality from the perspectives of colorfulness, sharpness, and contrast, while UCIQE reflects image quality by combining chroma, saturation, and luminance contrast. Higher values of both UIQM and UCIQE indicate better restored visual quality. These two metrics were used as the primary quantitative criteria for benchmark subsets such as UIEB Challenge-60 and EUVP330, where reference clean images were unavailable.

For paired synthetic evaluation, full-reference metrics were additionally used to measure restoration fidelity. Specifically, the peak signal-to-noise ratio (PSNR) and the structural similarity index measure (SSIM) were employed to evaluate pixel-level reconstruction quality and structural consistency, respectively. Higher PSNR and SSIM values indicate that the restored result is closer to the corresponding ground-truth reference image. These metrics complement UIQM and UCIQE by providing controlled quantitative evaluation under known blur conditions.

To evaluate the blur parameter estimation stage, the accuracy of the estimated blur length and blur angle was measured when ground-truth blur parameters were available. Let $L_{i}$ and $\theta_{i}$ denote the estimated blur length and blur angle of the *i*-th sample, and let $L_{i}^{*}$ and $\theta_{i}^{*}$ denote the corresponding ground-truth values. The mean absolute error (MAE) of blur length was defined as follows:

(15) $${\mathrm{MAE}}_{L}=\frac{1}{M}\sum_{i=1}^{M}\left|L_{i}-L_{i}^{*}\right|$$

where M is the number of evaluated samples. Since motion blur direction has angular periodicity, the blur angle error was measured by the minimum absolute angular difference:

(16) $${\mathrm{MAE}}_{\theta }=\frac{1}{M}\sum_{i=1}^{M}\min\left(\left|\theta_{i}-\theta_{i}^{*}\right|,\pi -\left|\theta_{i}-\theta_{i}^{*}\right|\right)$$

Lower values of ${MAE}_{L}$ and ${MAE}_{\theta }$ indicate more accurate blur parameter estimation. By combining no-reference restoration metrics, full-reference fidelity metrics, and parameter estimation accuracy, the evaluation protocol provides a more complete assessment of both the restoration performance and the physical reliability of the proposed framework.

For readability, the blur-angle errors reported in the experimental tables are converted from radians to degrees.

### 4.4. Objective Comparison on Real Underwater Benchmarks

Table 2 and Table 3 summarize the no-reference objective evaluation results on the UIEB Challenge-60 and EUVP330 datasets. Since clean reference images are unavailable for these real underwater benchmark subsets, evaluation was performed using the UIQM and UCIQE metrics introduced in Section 4.3. As shown in both tables, the proposed method achieves the best performance among the compared methods on both datasets.

On the UIEB Challenge-60 dataset, the proposed method achieves a UIQM score of 4.09 and a UCIQE score of 0.56, whereas UFPNet obtains 2.73 and 0.47, and Phaseformer obtains 2.93 and 0.52, respectively. On the EUVP330 dataset, the proposed method achieves a UIQM score of 3.40 and a UCIQE score of 0.58, compared with 1.93 and 0.46 for UFPNet, and 2.41 and 0.53 for Phaseformer. These results indicate that the proposed framework consistently improves both no-reference underwater image quality measures across different real underwater scenes.

Compared with the strongest comparison method, Phaseformer, the proposed method improves UIQM by 1.16 and UCIQE by 0.04 on UIEB Challenge-60, and improves UIQM by 0.99 and UCIQE by 0.05 on EUVP330. The consistent gains across both datasets suggest that incorporating physically estimated blur information into the attention mechanism benefits not only structural clarity, but also overall color and contrast consistency in restored underwater images. Since UIQM emphasizes sharpness, contrast, and colorfulness, and UCIQE reflects chroma and luminance-related image quality, the quantitative improvements are consistent with the design objective of the proposed PSF-guided restoration framework.

It should also be noted that the results reported in this subsection are based on no-reference evaluation of real underwater images. To provide a more complete assessment, full-reference evaluation on synthetically blurred underwater data and quantitative analysis of blur parameter estimation are further reported in the following subsections.

### 4.5. Full-Reference Evaluation on Synthetic Blurred Underwater Images

To complement the no-reference evaluation on real underwater benchmark images, full-reference experiments were conducted on synthetically blurred underwater images, where each degraded input had a corresponding sharp reference image. This evaluation provides controlled quantitative verification of the restoration fidelity of the proposed PSF-guided Transformer framework and complements the real-image benchmark results reported in Section 4.4.

The synthetic paired test set was constructed from paired underwater reference images using linear motion blur kernels with randomly sampled blur lengths and blur angles. In the default setting adopted in this study, the blur length was uniformly sampled from 5 to 25 pixels, and the blur angle was uniformly sampled from 0° to 180°. No additional noise was introduced unless otherwise stated. The synthetic test set was kept disjoint from the training data to ensure fair evaluation.

Because the blur kernels are known for the synthetic paired test set, two classical non-blind deblurring baselines, Wiener filtering and Richardson–Lucy deconvolution, were additionally included for comparison. Full-reference performance was evaluated using the peak signal-to-noise ratio (PSNR) and the structural similarity index measure (SSIM). Higher PSNR and SSIM values indicate better reconstruction quality and structural consistency.

Table 4 summarizes the full-reference evaluation results on the synthetic blurred underwater test set. As shown in the table, the proposed method achieves the best overall performance among all compared methods, obtaining a PSNR of 24.23 dB and an SSIM of 0.918. In comparison, Wiener filtering achieves 18.1 dB and 0.72, Richardson–Lucy deconvolution achieves 19.5 dB and 0.77, UFPNet achieves 20.15 dB and 0.818, and Phaseformer achieves 18.78 dB and 0.758, respectively.

Compared with the strongest learning-based baseline, UFPNet, the proposed method improves PSNR by 4.08 dB and SSIM by 0.100. Compared with Richardson–Lucy deconvolution, the proposed method improves PSNR by 4.73 dB and SSIM by 0.148. Compared with Wiener filtering, the proposed method improves PSNR by 6.13 dB and SSIM by 0.198. These results indicate that the proposed physically guided restoration framework outperforms both classical non-blind deconvolution methods and recent deep-learning-based baselines under controlled blur conditions.

These results indicate that the proposed physically guided restoration framework is effective not only in improving no-reference underwater image quality on real benchmark images, but also in recovering reference-consistent image structures under controlled blur conditions. In particular, the improvement in SSIM suggests that incorporating the estimated PSF into the attention mechanism helps preserve structural details more faithfully, while the gain in PSNR indicates improved pixel-level reconstruction accuracy.

Combined with the real-image no-reference evaluation in Section 4.4, the full-reference results demonstrate that the proposed PSF-guided Transformer framework achieves both perceptual improvement and reference-consistent restoration, thereby providing stronger evidence of its effectiveness for underwater motion deblurring.

### 4.6. Quantitative Evaluation of Blur Parameter Estimation

To further verify the effectiveness of the blur estimation stage described in Section 3.1, quantitative experiments were conducted on synthetically blurred underwater images with known blur parameters. Since the ground-truth blur length and blur angle were available for these synthetic samples, the accuracy of the proposed two-stage estimation strategy could be evaluated directly. This experiment was designed to assess whether the estimated blur parameters were sufficiently reliable to support subsequent PSF construction and PSF-guided restoration.

Following the synthetic data generation protocol described in Section 4.1, linear motion blur kernels with known blur lengths and blur angles were applied to underwater reference images. For each blurred image, three estimation settings were evaluated: (1) coarse estimation based only on ellipse fitting, (2) refined estimation based only on negative-peak analysis, and (3) the proposed full two-stage estimation strategy that combines coarse ellipse fitting and negative-peak refinement. The estimation accuracy was evaluated using the blur-length mean absolute error ${MAE}_{L}$ and the blur-angle mean absolute error ${MAE}_{\theta }$ defined in Section 4.3. For readability, the blur-angle errors reported in this subsection are expressed in degrees.

Table 5 summarizes the quantitative results of blur parameter estimation. As shown in the table, the proposed two-stage strategy achieves the lowest errors in both blur length and blur angle among the compared estimation settings. Specifically, coarse ellipse fitting yields a blur-length MAE of 2.9 and a blur-angle MAE of 7.9°, while negative-peak-only estimation achieves 2.3 and 6.2°, respectively. In contrast, the proposed two-stage estimation reduces the blur-length MAE to 0.8 and the blur-angle MAE to 1.6°, demonstrating a clear improvement over both single-stage alternatives.

Compared with coarse ellipse fitting, the proposed two-stage strategy reduces the blur-length error by 72.4% and the blur-angle error by 79.7%. Compared with negative-peak-only estimation, it reduces the blur-length error by 65.2% and the blur-angle error by 74.2%. These results indicate that coarse ellipse fitting provides a stable global estimate of blur orientation and blur extent, whereas negative-peak analysis supplies more localized refinement cues. By combining these two complementary sources of information, the proposed method achieves more accurate and more robust blur parameter estimation.

The reduction in ${MAE}_{L}$ demonstrates that the proposed refinement strategy improves estimation of blur extent, while the lower ${MAE}_{\theta }$ confirms that the final blur direction becomes more consistent with the ground-truth motion orientation. These results support the design of Section 3.1, where coarse structural estimation and local peak-based refinement are jointly used to obtain a reliable PSF for subsequent restoration. In other words, the benefit of the proposed framework does not arise only from the Transformer backbone, but also from the improved physical reliability of the blur estimation stage.

The quantitative blur estimation results also help explain the restoration gains reported in Section 4.4 and Section 4.5. Since the PSF-guided attention mechanism depends on the accuracy of the estimated blur prior, more accurate blur length and blur angle estimation directly improves the quality of the guidance signal injected into self-attention. Therefore, the improvements in restoration performance are consistent with the improved accuracy of the proposed two-stage blur parameter estimation process.

### 4.7. Subjective Evaluation

In addition to the quantitative results reported in Section 4.4, Section 4.5 and Section 4.6, subjective evaluation was conducted through qualitative visual comparison on representative examples from the UIEB Challenge-60 and EUVP330 datasets. The selected images contain various types of underwater degradation, including motion blur, low contrast, color distortion, and haze-like scattering. The proposed method was visually compared with two recent baseline methods, UFPNet and Phaseformer, in order to assess the perceptual quality of restored images under realistic underwater conditions.

**Figure 2:**
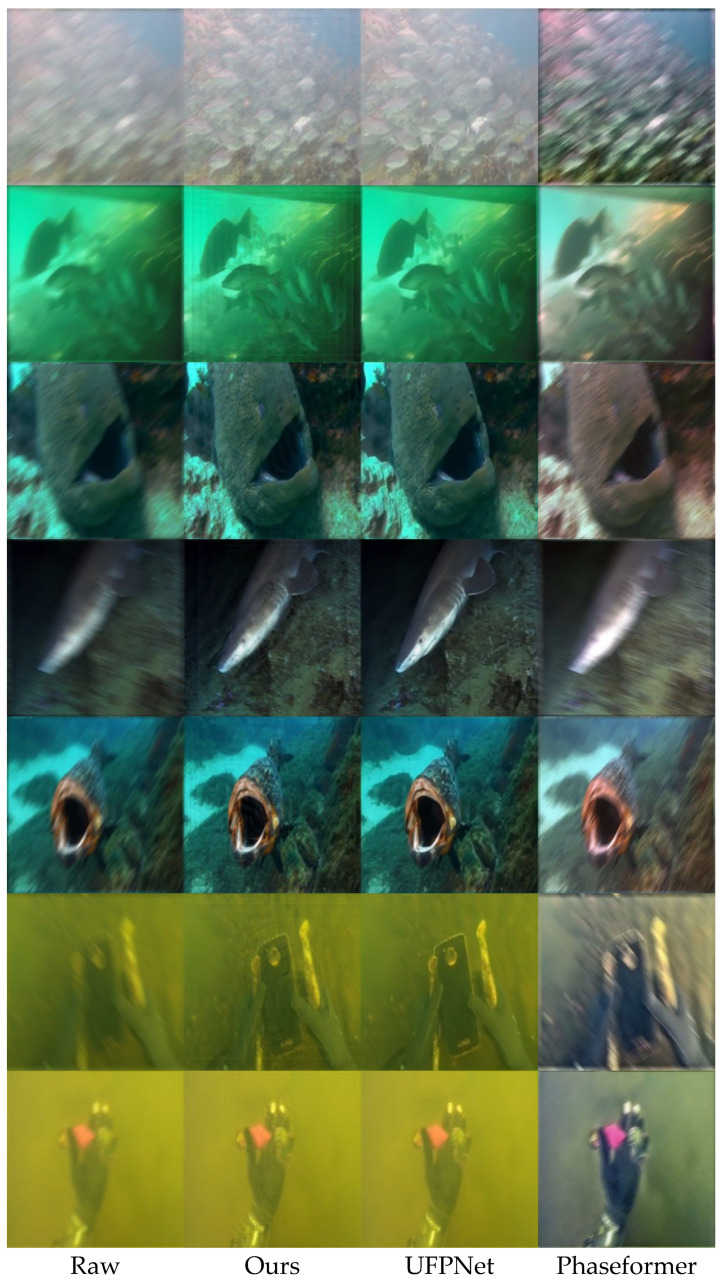
Visual comparison on the UIEB Challenge-60 dataset.

Figure 2 presents representative visual results on the UIEB Challenge-60 dataset. In the raw blurred images, motion blur significantly reduces structural clarity, making targets such as fish bodies, contours, and underwater structures difficult to distinguish. Both baseline methods partially suppress the degradation, but residual blur and limited detail recovery are still visible in many regions. In contrast, the proposed method restores clearer object boundaries and recovers more structural details. For example, in scenes containing schools of fish and close-up fish images, the proposed method produces sharper contours and improves the visibility of fine textures.

**Figure 3:**
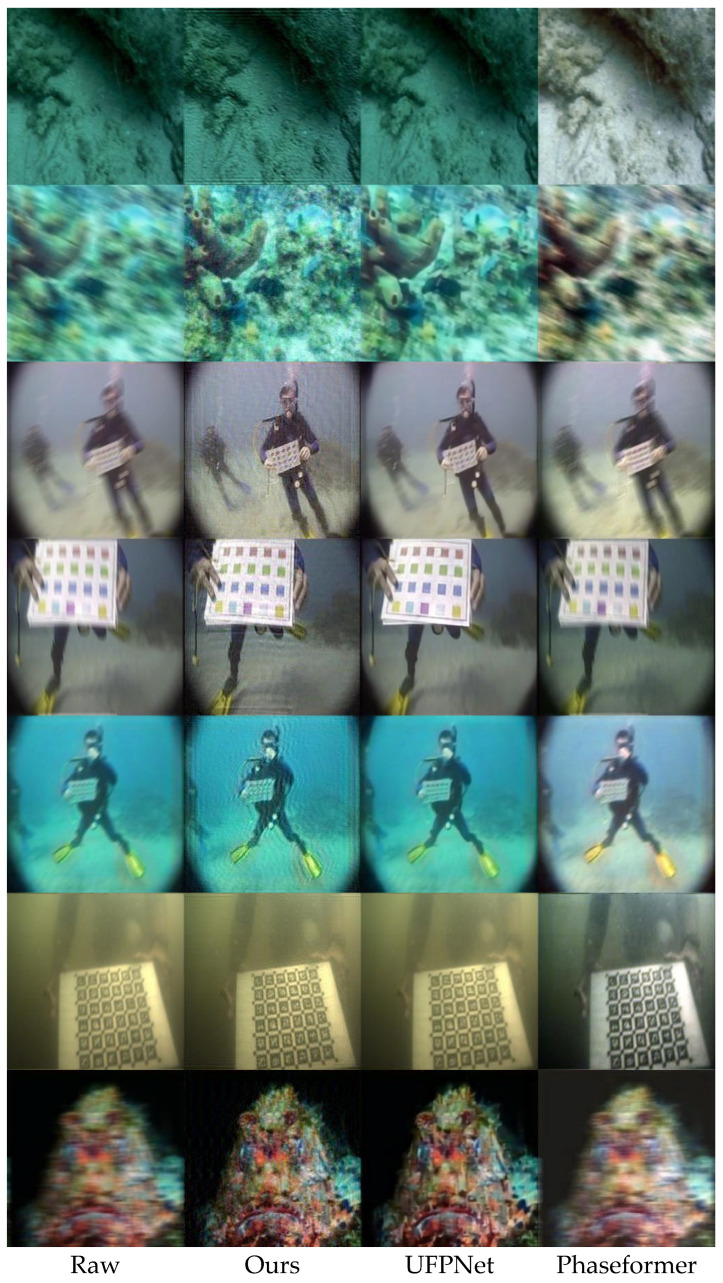
Visual comparison on the EUVP330 dataset.

Figure 3 shows additional qualitative comparisons on the EUVP330 dataset. Similar trends can be observed across scenes containing coral structures, rocks, and calibration charts. Compared with UFPNet and Phaseformer, the proposed method better preserves structural details, reduces residual blur artifacts, and maintains more consistent color representation. In particular, edges and repetitive patterns are more clearly recovered in the restored results of the proposed method, which is consistent with the objective of introducing blur-aware guidance into feature aggregation.

The qualitative observations are generally consistent with the quantitative trends reported in Section 4.4 and Section 4.5. Higher UIQM values are associated with stronger perceived sharpness and contrast, while higher UCIQE values reflect more stable chroma and luminance consistency. At the same time, it should be noted that no-reference metrics do not always fully capture local perceptual preferences. In some local regions, a baseline method may appear slightly sharper due to more aggressive local enhancement, but this can also be accompanied by residual artifacts or less stable global appearance. By contrast, the proposed method provides a better overall balance among blur removal, structural recovery, and underwater color consistency.

Overall, the subjective evaluation suggests that incorporating the PSF-guided attention mechanism improves the visual restoration of blurred underwater images. By introducing blur-related information into the attention computation, the proposed framework can better recover structural details in motion-degraded regions while maintaining more coherent image appearance. These observations are consistent with the design of the PSF-guided attention mechanism introduced in Section 3.

### 4.8. Ablation Study

To evaluate the contribution of the physical guidance component in the proposed framework, an ablation study was conducted on two representative underwater benchmark datasets: UIEB Challenge-60 and EUVP330. The purpose of this experiment was to isolate the effect of physically guided restoration by comparing the full model with a baseline Transformer restoration network using the same backbone but without the blur estimation and PSF-guided attention modules. In this way, the ablation focuses on the contribution of the physical-guidance pathway as a whole rather than on general backbone capacity.

Two model configurations were evaluated:

(1) **Baseline (Uformer):** the Transformer restoration backbone without the blur estimation stage and without PSF-guided attention.

(2) **Proposed model:** the complete framework incorporating two-stage blur estimation and PSF-guided attention.

The evaluation was performed using the no-reference underwater image quality metrics UIQM and UCIQE, which reflect perceptual quality in terms of sharpness, contrast, and color consistency. Table 6 summarizes the ablation results on the two benchmark datasets. As shown in the table, the proposed model consistently outperforms the baseline Uformer on both datasets and under both evaluation metrics.

On the UIEB Challenge-60 dataset, the proposed model achieves a UIQM score of 4.09, compared with 2.02 for the baseline Uformer, corresponding to an absolute improvement of 2.07. On the EUVP330 dataset, the proposed model achieves a UIQM score of 3.40, compared with 1.58 for the baseline, yielding an improvement of 1.82. Similar trends are observed for UCIQE: on Challenge-60, the proposed model achieves 0.56 versus 0.46 for the baseline, and on EUVP330 it achieves 0.58 versus 0.45, corresponding to absolute gains of 0.10 and 0.13, respectively.

These results indicate that incorporating the physically estimated blur prior substantially improves the restoration performance of the Transformer backbone. Without explicit blur guidance, the baseline Uformer can still improve image quality to some extent through data-driven feature learning, but it cannot adapt its feature aggregation to the sample-specific motion blur structure. In contrast, the proposed framework first estimates blur parameters from the input image, constructs the corresponding PSF, and then injects this physically meaningful guidance into the self-attention computation. This allows the network to better emphasize motion-degraded regions and to recover more consistent structures and color information.

The ablation results are also consistent with the full-reference results in Section 4.5 and the qualitative comparisons in Section 4.7. Together, these observations suggest that the performance gain of the proposed framework does not arise solely from using a Transformer backbone, but from combining physically estimated blur cues with attention-based feature aggregation. Therefore, the ablation study supports the central claim of this work: physically guided attention is an effective mechanism for underwater motion deblurring.

### 4.9. Computational Cost Analysis

To evaluate the computational characteristics of the proposed framework, we compared the model complexity and inference efficiency of different learning-based methods at an input resolution of 256 × 256. Table 7 reports the number of parameters, FLOPs, and average inference time per image. For the proposed method, the reported inference time includes both the blur estimation stage and the restoration network.

As shown in Table 7, the proposed method has a computational cost comparable to that of recent Transformer-based restoration methods while remaining slower than the lighter baseline UFPNet. This overhead is expected because the proposed framework introduces an additional blur estimation stage and PSF-guided attention. Nevertheless, the model remains practical for offline and near-real-time underwater restoration.

### 4.10. Real-World Water-Tank Validation

**Figure 4:**
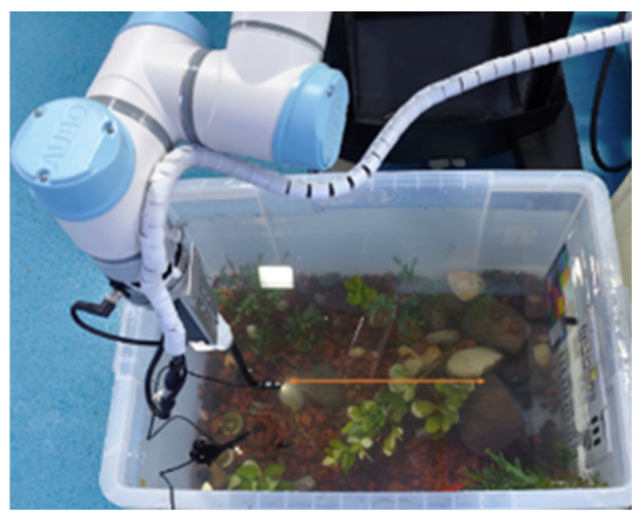
Water-tank experimental platform. The orange line indicates the manipulator motion path.

To further evaluate the practical applicability of the proposed framework, real-world experiments were conducted on the controlled water-tank platform described in Section 4.1. As is shown in Figure 4, the tank environment was arranged with stones, aquatic plants, and sand to simulate weak-texture underwater scenes similar to seabed environments. A monocular camera was mounted on a motion platform and used to capture image sequences during controlled linear and rotational motion, thereby generating realistic underwater motion blur. Water turbidity and illumination were adjusted by introducing suspended particles and modifying the lighting configuration, which enabled the acquisition of underwater images under different visibility and blur conditions.

To quantitatively evaluate the restoration performance under real underwater conditions, the proposed method was compared with UFPNet and Phaseformer on water-tank image sequences captured at camera motion speeds ranging from 2 cm/s to 10 cm/s. Since clean reference images were unavailable for these real-world sequences, evaluation was performed using the no-reference underwater image quality metrics UIQM and UCIQE. Table 8 reports the UIQM comparison results, while Table 9 reports the UCIQE comparison results.

As shown in Table 8, the proposed method consistently outperforms UFPNet across all tested motion speeds and remains competitive with, and often superior to, Phaseformer. In particular, the proposed method achieves the highest UIQM values at 2 cm/s, 3 cm/s, 8 cm/s, 9 cm/s, and 10 cm/s, with scores of 5.55, 5.02, 5.75, 5.04, and 4.80, respectively. At intermediate speeds from 4 cm/s to 7 cm/s, the proposed method remains very close to Phaseformer, with only small differences. These results indicate that the proposed framework improves structural clarity and perceptual quality in realistic underwater motion-blurred scenes while maintaining stable performance under varying motion conditions.

The UCIQE results in Table 9 show a similarly stable trend. The proposed method achieves 0.59 or 0.60 at most tested motion speeds and outperforms UFPNet consistently. Compared with Phaseformer, the proposed method either matches or slightly exceeds the baseline at all tested speeds. This suggests that the restored images maintain consistent chroma and luminance-related quality even when the blur level changes with camera motion speed. Taken together, the water-tank experiments provide additional real-world evidence that the proposed PSF-guided attention mechanism improves underwater motion deblurring beyond benchmark datasets and synthetic evaluation.

## 5. Discussion

The proposed physically guided Transformer framework for underwater motion deblurring demonstrates that injecting an explicitly estimated point spread function (PSF) as an image-dependent guidance term into Transformer self-attention can substantially and consistently improve restoration performance. This conclusion is strongly supported by the ablation study in Table 6: once the physical-guidance pathway is removed, the baseline Uformer exhibits a dramatic performance drop, with UIQM on UIEB Challenge-60 decreasing from 4.09 to 2.02, i.e., by more than 50%. Such a pronounced gap indicates that, although a purely data-driven Transformer can model long-range dependencies, its attention mechanism does not naturally align itself with the directional structure of motion blur in weak-texture and mixed-degradation underwater scenes. By contrast, the proposed method first obtains reliable blur length and blur angle estimates through two-stage cepstrum-based estimation (Table 5: ${MAE}_{L}=0.8$, ${MAE}_{\theta }=1.6{}^{\circ}$), and then encodes this physical prior as a spatial bias matrix that directly modulates attention affinity. This design offers two major advantages. First, it explicitly informs the network of the extent and orientation of the underlying blur, allowing the model to preferentially aggregate information along the blur direction during restoration. Second, because the PSF is estimated independently for each input image, the guidance signal is sample-adaptive. This is fundamentally different from both classical non-blind deconvolution with a fixed kernel and purely data-driven methods that only learn implicit blur patterns from training data. This also helps explain why the proposed method remains stable in the real-world water-tank experiments: even when the camera motion speed varies from 2 cm/s to 10 cm/s, the restored image quality remains consistently high, with UCIQE staying within the narrow range of 0.58–0.60.

The present study further evaluates the proposed framework from the perspectives of generalization and robustness, which together underpin its practical utility. Generalization is reflected in the transfer from synthetic training data to real underwater images. Although the model is trained only on synthetically blurred underwater images generated from reference underwater images and random motion kernels, it achieves the best no-reference performance on two distinct real underwater benchmarks, namely UIEB Challenge-60 and EUVP330. In contrast, the performance of UFPNet and Phaseformer decreases substantially on these real datasets. For example, on EUVP330, UIQM drops from 3.40 for the proposed method to 1.93 for UFPNet. This suggests that the physically guided mechanism enables the model to learn a blur-formation prior that is less dependent on the training data distribution, rather than merely overfitting to the specific degradation patterns of the synthetic training set. Robustness is further examined in the controlled water-tank experiment with varying motion speeds. When the motion speed, and therefore the blur length, changes continuously from 2 to 10 cm/s, the proposed method maintains both high absolute image quality and stable quantitative behavior, with standard deviations of approximately 0.36 for UIQM and 0.008 for UCIQE. Although UFPNet shows a smaller numerical variance, this is mainly because its outputs remain consistently poor across all tested speeds. By contrast, the proposed method is able to preserve substantially better restoration quality while remaining stable under changing motion conditions. This result indicates that the estimated PSF can reliably track changes in real motion parameters and provide a stable guidance signal to the restoration network.

On the synthetic blurred underwater test set, the proposed method outperforms both classical non-blind deconvolution methods (Wiener filtering and Richardson–Lucy) and recent deep baselines (UFPNet and Phaseformer). Notably, the classical non-blind methods still achieve only about 18–19 dB PSNR in Table 4, even when the blur kernels are known, which remains far below the 24.23 dB achieved by the proposed method. This reveals an important fact: in underwater imaging with complex mixed degradations, accurate kernel estimation followed by direct deconvolution is not sufficient, because the deconvolution process tends to amplify noise and scattering-related artifacts. Instead, the proposed method integrates the PSF into an end-to-end Transformer as a soft guidance signal, thereby avoiding the adverse side effects of explicit deconvolution while exploiting the representation power of deep restoration to compensate for residual errors in blur estimation. Compared with underwater enhancement methods such as Phaseformer, the proposed method explicitly models the directional nature of motion blur, which explains why its advantage is especially evident in motion-blur-dominant scenes. At the same time, in some images where color distortion or haze-like scattering dominates and motion blur is relatively mild, the visual difference between the proposed method and Phaseformer becomes smaller, as can be observed in Figure 2 and Figure 3. This suggests that a single motion-blur prior is not sufficient to handle all types of underwater degradation, and that richer physical guidance will be needed in future work.

### Limitations

Despite the encouraging results, several limitations should be acknowledged.

First, the current framework assumes that motion blur is approximately spatially uniform within the processed image crop. This assumption is reasonable for the present synthetic setting and for most images in the controlled water-tank experiment, but in real underwater robotic operations, non-uniform blur is often more common due to camera rotation, scene-depth variation, and dynamic objects. The current PSF-guided design is not yet capable of modeling pixel-wise or region-wise spatially varying blur fields.

Second, the proposed method explicitly models only motion blur and does not yet incorporate other physical priors such as scattering, absorption, or defocus blur. In mixed-degradation scenarios where motion blur is not dominant, the benefit of a single motion-blur PSF prior may therefore be limited. For example, in some strongly color-shifted images from EUVP330, the gain in UCIQE is smaller than the gain in UIQM, suggesting that color restoration in such cases still depends more strongly on the data-driven component than on the current physical guidance mechanism.

Third, the current model is computationally more expensive than the compared baselines. The proposed method requires approximately 85 ms per image at 256 × 256, compared with 55 ms for Phaseformer and 30 ms for UFPNet. Although this is acceptable for offline restoration and near-real-time applications, it is still less suitable for deployment on resource-constrained embedded underwater robotic platforms, where both computation and power budgets are limited.

Fourth, although the two-stage blur estimation process significantly reduces blur-parameter error (${MAE}_{\theta }=1.6{}^{\circ}$), residual estimation errors may still propagate into the PSF and subsequently influence attention guidance. At present, the framework does not include an explicit uncertainty-aware mechanism to down-weight unreliable guidance when the PSF estimate is less accurate.

The central contribution of this study is to demonstrate that an explicit and physically interpretable blur prior can serve as an effective inductive bias in deep restoration networks, thereby compensating for the limitations of purely data-driven approaches in weak-texture and dynamic underwater environments. The proposed two-stage cepstrum-based estimation and PSF-guided attention mechanism achieve superior perceptual quality, restoration fidelity, and robustness across multiple datasets and real-world experiments. Despite limitations such as the assumption of spatially uniform blur and the current computational cost, this work establishes a feasible technical pathway for integrating physical priors with data-driven learning in underwater image restoration and provides clear directions for future improvement.

## 6. Conclusions

This paper presented a physically guided Transformer-based framework for underwater motion deblurring. The proposed method combines two-stage cepstrum-based blur parameter estimation with a PSF-guided attention mechanism, enabling the restoration network to integrate sample-specific blur information into feature aggregation. Unlike purely data-driven restoration approaches, the proposed framework explicitly estimates blur length and blur angle from the degraded input image, constructs the corresponding PSF, and injects this physical prior into Transformer self-attention during restoration. The experimental results on public underwater benchmarks, synthetic blurred underwater images, and controlled water-tank image sequences demonstrate the effectiveness of the proposed approach. On UIEB Challenge-60 and EUVP330, the proposed method achieves the best UIQM and UCIQE scores among the compared methods. On the synthetic blurred underwater test set, it also achieves the best full-reference performance, reaching 24.23 dB PSNR and 0.918 SSIM. In addition, the proposed two-stage blur estimation strategy reduces the blur-length MAE to 0.8 and the blur-angle MAE to 1.6°, confirming the physical reliability of the guidance signal used for restoration. Together, these results show that physically guided attention can improve both restoration fidelity and perceptual quality in blurred underwater images, and that the performance gain of the proposed framework arises not only from the Transformer backbone, but also from the integration of explicit blur estimation and PSF-guided attention under practical underwater imaging conditions.

Future work will extend the present framework in three directions. First, the current uniform-PSF model will be generalized to a spatially varying blur field in order to handle non-uniform motion blur. Second, additional physical priors, such as scattering and absorption cues, will be incorporated to build a more comprehensive physically guided restoration framework. Third, through model lightweighting and embedded deployment, the proposed method will be further adapted for practical underwater robotic applications, such as visual navigation, object detection, and related downstream perception tasks.

## Figures and Tables

**Table 1:** Summary of representative related work on motion deblurring and underwater restoration.

Method	Category	Physical Prior	Explicit Blur Estimation	Underwater-Specific	Main Strength	Main Limitation
Wiener filtering [6]	Classical non-blind deblurring	Yes	No	No	Simple and interpretable	Requires known kernel; sensitive to mismatch and noise
Richardson–Lucy [21]	Classical non-blind deblurring	Yes	No	No	Effective iterative deconvolution	May amplify artifacts and noise without careful regularization
Radon/Cepstrum-based methods [23,24,25,26,27]	Classical non-blind deblurring	Yes	Yes	No	Interpretable blur-angle and blur-length estimation	Unstable under weak texture and low contrast
MIMO-UNet [12]	CNN deblurring	No	No	No	Strong end-to-end restoration	Mainly local feature aggregation
Restormer/Uformer/MPRFormer [13,14,15]	Transformer restoration	No	No	No	Long-range dependency modeling	Blur learned implicitly
UFPNet [28]	Deep blind deblurring	Partial	Implicit	No	Long-range dependency modeling	Not underwater-specific
Phaseformer [29]	Underwater restoration	No	No	Yes	Good underwater restoration performance	Does not explicitly model motion blur formation
Ours	Physically guided Transformer	Yes	Yes	Yes	Good underwater restoration performance	Currently assumes approximately uniform blur within each crop

**Table 2:** Objective comparison on the UIEB Challenge-60 dataset in terms of UIQM and UCIQE.

Challenge-60			
Method	Ours	UFPNET	Phaseformer
UIQM	4.09	2.73	2.93
UCIQE	0.56	0.47	0.52

**Table 3:** Objective comparison on the EUVP330 dataset in terms of UIQM and UCIQE.

EUVP330			
Method	Ours	UFPNET	Phaseformer
UIQM	3.40	1.93	2.41
UCIQE	0.58	0.46	0.53

**Table 4:** Full-reference comparison on the synthetic blurred underwater test set in terms of PSNR and SSIM. Higher values indicate better performance.

Method	Ours	UFPNET	Phaseformer	Wiener	Richardson–Lucy
PSNR	24.23	20.15	18.78	18.1	19.5
SSIM	0.918	0.818	0.758	0.72	0.77

**Table 5:** Quantitative evaluation of blur parameter estimation on the synthetic blurred underwater test set in terms of blur-length MAE and blur-angle MAE. Lower values indicate better estimation accuracy. Blur-angle errors are reported in degrees for readability.

Method	Coarse Ellipse Fitting	Negative-Peak Only	Two-Stage Estimation (Ours)
MAE_L_	2.9	2.3	0.8
MAE_θ_	7.9°	6.2°	1.6°

**Table 6:** Ablation study of the proposed physical-guidance mechanism on the UIEB Challenge-60 and EUVP330 datasets in terms of UIQM and UCIQE. Higher values indicate better performance.

	Challenge-60 UIQM	Challenge-60 UCIQE	EUVP330 UIQM	EUVP330 UCIQE
Proposed	4.09	0.56	3.40	0.58
Uformer	2.02	0.46	1.58	0.45

**Table 7:** Computational cost comparison of different learning-based methods at 256 × 256 input resolution. Lower inference time indicates better efficiency.

	Params (M)	FLOPs (G)	Inference Time (ms/Image)
UFPNet	18	35	30
Phaseformer	24	60	55
Ours	30	85	85

**Table 8:** UIQM comparison on real-world water-tank image sequences under different camera motion speeds. Higher values indicate better performance.

Speed (cm/s)	UFPNET	Ours	Phaseformer
2	3.59	5.55	5.08
3	3.65	5.02	5.00
4	3.60	4.87	4.88
5	3.65	4.66	4.75
6	3.59	4.81	4.85
7	3.63	5.00	5.03
8	3.58	5.75	4.85
9	3.59	5.04	4.91
10	3.58	4.80	4.79

**Table 9:** UCIQE comparison on real-world water-tank image sequences under different camera motion speeds. Higher values indicate better performance.

Speed (cm/s)	UFPNET	Ours	Phaseformer
2	0.41	0.59	0.59
3	0.40	0.60	0.59
4	0.40	0.59	0.58
5	0.41	0.58	0.57
6	0.40	0.59	0.58
7	0.40	0.59	0.58
8	0.41	0.59	0.58
9	0.40	0.58	0.58
10	0.41	0.58	0.58

## Data Availability

This study used publicly accessible underwater image datasets. The UIEB dataset is freely available at https://li-chongyi.github.io/proj_benchmark.html (accessed on 22 April 2026) and the EUVP dataset at https://irvlab.cs.umn.edu/resources/euvp-dataset (accessed on 22 April 2026). No additional data were created or analyzed beyond these existing resources.
